# The epidemiology of personality disorders in the Sao Paulo Megacity general population

**DOI:** 10.1371/journal.pone.0195581

**Published:** 2018-04-24

**Authors:** Geilson Lima Santana, Bruno Mendonca Coelho, Yuan-Pang Wang, Alexandre Dias Porto Chiavegatto Filho, Maria Carmen Viana, Laura Helena Andrade

**Affiliations:** 1 Section of Psychiatric Epidemiology, Institute & Department of Psychiatry (LIM-23), University of Sao Paulo Medical School, Sao Paulo, SP, Brazil; 2 Department of Epidemiology, University of Sao Paulo Public Health School, Sao Paulo, SP, Brazil; 3 Department of Social Medicine, Federal University of Espirito Santo, Vitoria, ES, Brazil; Humboldt-Universitat zu Berlin Mathematisch Naturwissenschaftliche Fakultat, GERMANY

## Abstract

**Introduction:**

Most studies on the epidemiology of personality disorders (PDs) have been conducted in high-income countries and may not represent what happens in most part of the world. In the last decades, population growth has been concentrated in low- and middle-income countries, with rapid urbanization, increasing inequalities and escalation of violence. Our aim is to estimate the prevalence of PDs in the Sao Paulo Metropolitan Area, one of the largest megacities of the world. We examined sociodemographic correlates, the influence of urban stressors, the comorbidity with other mental disorders, functional impairment and treatment.

**Methods:**

A representative household sample of 2,942 adults was interviewed using the WHO-Composite International Diagnostic Interview and the International Personality Disorder Examination-Screening Questionnaire. Diagnoses were multiply imputed, and analyses used multivariable regression.

**Results and discussion:**

Prevalence estimates were 4.3% (Cluster A), 2.7% (Cluster B), 4.6% (Cluster C) and 6.8% (any PD). Cumulative exposure to violence was associated with all PDs except Cluster A, although urbanicity, migration and neighborhood social deprivation were not significant predictors. Comorbidity was the rule, and all clusters were associated with other mental disorders. Lack of treatment is a reality in Greater Sao Paulo, and this is especially true for PDs. With the exception of Cluster C, non-comorbid PDs remained largely untreated in spite of functional impairment independent of other mental disorders.

**Conclusion:**

Personality disorders are prevalent, clinically significant and undertreated, and public health strategies must address the unmet needs of these subjects. Our results may reflect what happens in other developing world megacities, and future studies are expected in other low- and middle-income countries.

## Introduction

The epidemiology of personality disorders (PDs) in the general population was largely unclear and speculative until the 1990s [1]. This landscape began to change with the publication of DSM-III [2] and its explicit diagnostic criteria and multiaxial classification system; the development of psychometrically-sound instruments and structured and semi structured clinical interviews; and the adoption of representative sampling techniques. Now it is known that the community prevalence of personality disorders in adults varies between 4.4 and 13.4% [3–9]. Most of these surveys, however, have been conducted in high-income countries and may not reflect what happens in most part of the world. There is a paucity of data coming from less developed regions, despite their being home to 83% of the world population [10].

According to the United Nations [11], the world has faced a remarkable urbanization in the last decades. This process has taken place predominantly in low- and middle-income countries (LMIC), which contained, in 2014, twenty-three of the 29 megacities with more than 10 million inhabitants. Projections indicate this is an ongoing process. By 2030, the world is expected to have 41 megacities, and more than 90% of the future urban population growth will be in LMIC.

The Sao Paulo Metropolitan Area (SPMA), situated in southeast Brazil, is one of the largest megacities of the world, with approximately 20.5 million inhabitants [12]. In spite of its historical, cultural, and economic specificities, it shares many common characteristics with other LMIC megacities and may be illustrative of what happens in different parts of the developing world.

The SPMA had a pivotal role in the Brazilian industrialization during the 20^th^ century. This fast and concentrated process was associated with intense internal migration from rural and poorer regions, resulting in fast-paced, unplanned urban growth [13, 14]. As seen in other metropolitan areas, infrastructure and services were insufficient and inadequate to absorb this rapidly increasing population. Because of housing shortage, there was inordinate land occupation, leading to the emergence and growth of slums. The informal work sector expanded, with aggravation of social and economic inequalities [15, 16]. Violence escalated and insecurity spread over the metropolitan region [17].

Being born and raised in urban areas or even living in a city at a given moment in time may have deleterious effects on mental health [16, 18–23]. Much remains to be explored, however, about the impact of urban stressors on personality disorders.

**Fig 1:**
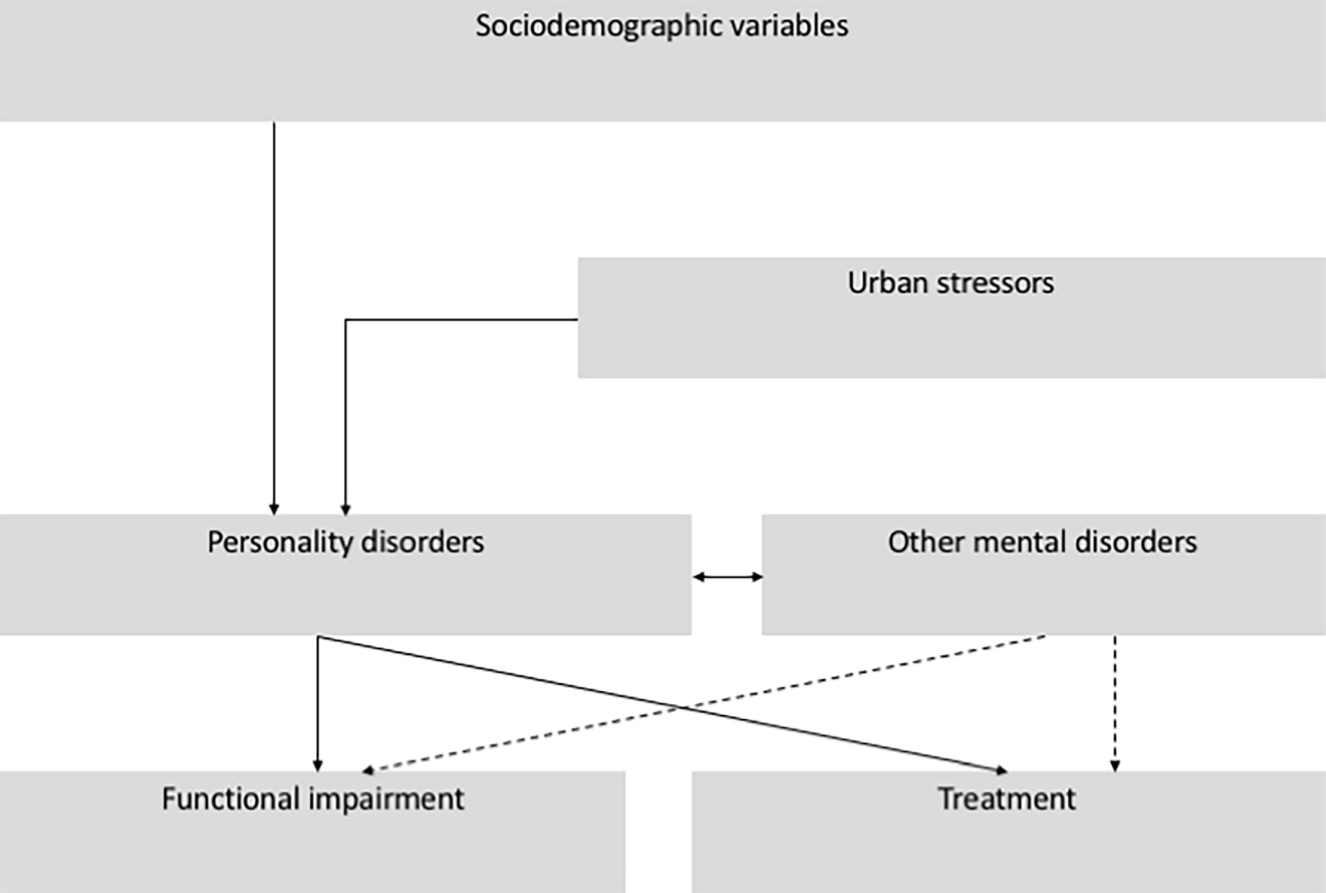
Conceptual framework of the study.

To our knowledge, there is no study on the epidemiology of PDs in the Brazilian general population. The main goal of this study is to help fill this gap and contribute to expand the scarce literature from less developed regions. In other words, our aims are: i. to describe the prevalence of personality disorders in the SPMA; ii. to explore the influence of sociodemographic variables on PDs; iii. to evaluate the impact of urban stressors—such as urbanicity, migration, neighborhood social deprivation and exposure to violence—on personality pathology; iv. to examine the co-occurrence of personality and other mental disorders in the SPMA; v. to explore the impact of PDs on functional impairment; vi. and to assess the use of health services, adjusting for the effect of other mental disorders (Fig 1).

## Materials and methods

### Sample

Data are from the Sao Paulo Megacity Mental Health Survey (SPMHS), the Brazilian branch of the World Mental Health Survey Initiative (WMH) [24]. SPMHS is a cross-sectional study of psychiatric morbidity with a representative sample of the adult population living in the Sao Paulo Metropolitan Area, Brazil [25]. This region is comprised of the city of Sao Paulo and its 38 surrounding municipalities, and at the time of data collection (2005–2007), 11 million inhabitants were 18 years or older [26].

Respondents were selected by means of a stratified, multistage area probability sample of households. In each household, the interviewer obtained a list of all residents, with information on age, gender and family relationship to the informant. This list was then sorted by gender and inverse order of age, and the eligible respondents were identified, i.e., those who were 18 years or older, Portuguese-speaking and without any disability or handicap that would impair their ability to participate in the study. One resident was then randomly selected by means of a Kish grid, a probabilistic method for selecting household respondents from a table of random numbers [27]. In addition, in a random 20% sample of households where the selected respondent was married or living as married, the spouse was identified and selected for interview. SPMHS procedures were approved by the Ethical and Research Committee of the University of Sao Paulo Medical School. Respondents were interviewed only after written informed consent was obtained and total confidentiality was assured. A total of 5,037 subjects were evaluated, with a global response rate of 81.3%. Detailed descriptions of sampling are presented elsewhere [28].

### Assessment

Face-to-face interviews, conducted by trained professional personnel, were comprised of two parts. Part I was administered to all 5,037 respondents and evaluated demographic characteristics and core mental disorders (major depression, dysthymia, bipolar I or II, panic disorder, specific phobia, social phobia, agoraphobia, generalized anxiety disorder (GAD), adult separation anxiety, substance use disorders, intermittent explosive disorder (IED), attention-deficit/hyperactivity disorder (ADHD), oppositional-defiant disorder (ODD) and conduct disorder). It also assessed suicidal behaviors, daily functioning and physical morbidity. Part II, in its turn, assessed personality disorders and a few additional conditions (posttraumatic stress disorder (PTSD) and obsessive-compulsive disorder (OCD)), along with risk factors, consequences and other correlates. It was administered to 2,942 subjects, including all those who met lifetime criteria for Part I disorders and a probability subsample of other respondents. Part II respondents are the focus of the current report.

### Measures

#### Personality disorders

Personality disorders in DSM-IV are grouped into three clusters, based on descriptive similarities: Cluster A (the “odd or eccentric” group, including paranoid, schizoid and schizotypal PDs), Cluster B (the “dramatic, emotional or erratic” group, including antisocial, borderline, histrionic and narcissistic PDs), and Cluster C (the “anxious or fearful” group, including avoidant, dependent and obsessive-compulsive PDs) [29].

Following the procedures adopted by the WMH [30], personality disorders were assessed with 33 screening questions from the International Personality Disorder Examination (IPDE) [31, 32]. These items were shown to be significant predictors of one or more of the DSM-IV personality disorders Clusters (A, B and C) or the overall diagnosis of any personality disorder (including personality disorder not otherwise specified) assessed by a clinician-administered IPDE [33, 34], as described in more detail later in the analysis subsection.

#### Other mental disorders

The presence of other mental disorders in the previous twelve months was assessed using the WMH version of the Composite International Diagnostic Interview (WMH-CIDI) [35]. This is a fully structured lay interview that yields diagnoses according to DSM-IV criteria. The instrument was translated and adapted to the Brazilian-Portuguese language [36]. In this report, we considered the following four classes of psychopathology: anxiety disorders (panic disorder, specific phobia, social phobia, agoraphobia without panic disorder, GAD, adult separation anxiety, PTSD and OCD); mood disorders (major depressive disorder, dysthymia, bipolar I or II); impulse-control disorders (conduct disorder, ODD, ADHD and IED); and substance use disorders (alcohol and drug abuse and dependence). Organic exclusion rules were used in making the diagnoses.

#### Sociodemographic correlates

Sociodemographics included gender, age at interview (standardized to a mean of zero and variance of 1.0), education (standardized the same way as age), employment status (employed/student/homemaker, retired, or other—i.e., unemployed or disabled), income (standardized the same way as age and education), and marital status (married or cohabiting, previously married, never married).

#### Exposure during early years of the life course to urban environment (urbanicity)

Respondents were asked whether they had been raised (i.e., spent most of their childhood and adolescence) in a large city or its suburbs, a small town or village, or a rural area. Three dummy variables were created to reflect levels of exposure to urbanicity [16]. Being raised in rural areas was considered the lower exposure level; being raised in a small town or village, the medium exposure level; and being raised in large cities, the highest exposure level.

#### Migration status

Respondents were asked if they were born outside the SPMA.

#### Neighborhood social deprivation (NSD) level

An index of NSD level developed by the Center for Metropolitan Studies [15] was assigned to each census unit to reflect social conditions in the SPMA geographical space using data from the 2000 Census [26]. This index is derived from a combination of socioeconomic deprivation dimensions (income, level of education, family size and percentage of families headed by a woman with low education level) and the population’s age structure.

#### Exposure to crime-related traumatic events

Seven crime-related traumatic events were selected from the list of experiences in the PTSD section of CIDI: kidnapped or held captive; “quicknapping” (a short-term kidnapping); stalked; mugged or threatened with a weapon; witnessed anyone being injured or killed, or unexpectedly saw dead body; witnessed atrocities or carnage; witnessed a close person being kidnapped, tortured or raped.

#### Impairment

The World Health Organization Disability Assessment Scale (WHO-DAS) [37] was used to evaluate impairment over a 30-day recall period in basic activities (self-care, mobility, cognition) and instrumental activities (quality of productive role performance, quality of social role performance). Each WHO-DAS dimension is scored on a 0–100 scale, where 0 represents no impairment and 100 represents complete impairment. We also estimated global functioning as the mean of the five domains of functioning. To facilitate interpretation, WHO-DAS scores were standardized to a mean of zero and variance of 1.0.

#### Treatment

Respondents were asked about treatment in the last year for problems with their emotions, “nerves” or substance use by a psychiatrist, other mental health professional (e.g., clinical psychologist, psychiatric social worker), a general medical healthcare provider, a human services professional (e.g., religious counsellor, social worker seen in a social service agency), or in the complementary-alternative medicine (CAM) sector (either receiving treatment by a CAM professional or participating in a self-help group). A visual list of provider types was presented to respondents when asking this question.

### Data analysis

Traditionally, the use of screening questionnaires to generate diagnoses involves a clinical calibration study where the screening instrument is compared to the gold standard diagnostic procedure (generally a structured or semi structured interview conducted by trained clinicians). Validity of a test may be defined as the ability to distinguish between those who have a disease and those who do not. Validity has two components: sensitivity (the ability to correctly identify those who have the disease), and specificity (the ability to correctly identify those who do not have the disease) [38]. A Receiver Operator Characteristic (ROC) Curve may be plotted to compare diagnoses obtained by the screening questionnaire and the gold standard diagnostic procedure. Then, it is possible to select an appropriate cut-off point for the screening instrument considering a good balance between sensitivity and specificity [39].

WMH, however, adopted an alternative procedure to generate diagnoses of Clusters A, B or C or any PD from responses to the screening questionnaire.

In the US National Comorbidity Survey Replication (NCS-R), which was the prototypical study of the WMH project, all Part II respondents (n = 5,692) answered the PD screening questionnaire. Responses to these questions were then combined to generate diagnoses based on a calibration study with a probability subsample (n = 214) of Part II respondents, oversampling those who screened positive [3]. In brief, clinical reappraisal interviews with the full IPDE were carried out by telephone by a veteran and well-trained clinician, blind to the screening responses. These interviews were tape recorded for quality control, and an experienced IPDE supervisor monitored the recordings and gave feedback to prevent drift. Prior research had shown that the IPDE generates valid personality disorder diagnoses when administered by telephone [40].

The next step was to link screening responses with the IPDE clinical diagnoses of Clusters A, B, C and any PD. Instead of establishing cutoff points to the screening questionnaire to estimate diagnoses, NCS-R researchers opted for using multiple imputation (MI), a method that offers approximately unbiased estimates taking uncertainty into account [41, 42]. In other words, multiple imputation provides statistically valid inferences in the context of values missing completely at random, as is the case of planned missingness [43–45], a strategy adopted by all WMH participant sites, including the Sao Paulo Megacity survey.

For the multiple imputation, ten prediction equations were created for each of the four diagnoses. Predictors included personality screening items, sociodemographic variables and questions related to other mental disorders. Final models were obtained by backward elimination of non-significant predictors. Ten predicted probabilities for each of the four PD diagnoses were then assigned to each respondent and were used to create ten multiple imputation datasets.

Prediction accuracy in the calibration sample was excellent in all the equations, with areas under the ROC Curves of 0.94 for Cluster A, 0.92 for Cluster B, 0.90 for Cluster C and 0.88 for any PD [3].

In the present study, we imputed PD diagnoses from IPDE screening questions using these prediction equations obtained in the US clinical reappraisal study, a procedure adopted by the WMH Survey Initiative [30].

The prevalence of PD Clusters and the regression coefficients were estimated by multiple imputation. This process followed three steps. In the first, the imputation phase, ten complete datasets were generated for each personality diagnosis (Clusters A, B, C and any PD). In the second step, analyses were carried out separately on each dataset, resulting in ten sets of parameter estimates (e.g., prevalence estimates). In the final step, the resulting ten sets of estimates were averaged to obtain a best estimate of the parameter, and coefficients and standard errors were adjusted for the variability between imputations according to Rubin’s combination rules [41] (S1 Fig).

Since values of the areas under the ROC Curve are high, these imputations are considered accurate and the precision of the parameter estimates approach the precision that would be achieved if personality disorders were directly assessed with IPDE interviews in the total sample [30].

Statistical analyses were performed in six steps. First, we estimated the prevalence of PDs. Then we assessed the association of PDs with sociodemographic variables in bivariate logistic regression models. Next step, we examined the influence of urban stressors on PDs. In order to account for the non-independence of the observations found by a previous study on this sample [46], and the fact that the variable NSD was measured on the contextual level (census tracts), the associations of PDs with urban stressors was initially analyzed by means of logistic multilevel models. Individuals were considered the first level, and the census tracts, the second level. However, intraclass correlation coefficients were zero (not shown) and we decided to present a simpler multivariable logistic model. Following that, we explored the co-occurrence of PDs and other mental disorders by estimating the prevalence of these co-occurrences and by means of logistic regression. After that, associations of PDs with impairment were estimated with linear regression equations adjusted for gender and age in the first model, and for gender, age and any other 12-month mental disorder in the second model. Finally, we estimated treatment prevalence among those with PDs and analyzed the association of PDs with treatment by means of logistic regression. The first model adjusted for gender and age, and the second model, for gender, age and any other 12-month mental disorder.

Logistic regression coefficients were exponentiated and reported as odds ratios (OR), along with their 95% confidence intervals (95% CI).

All analyses were conducted with Stata 15 [47]. Due to the complex sample design, imputed parameters were estimated using the “*mi estimate*: *svy*:*”* command [48], accounting for stratification, clustering and weighting. Concisely, data were weighted to adjust for the probabilities of selection and non-response in households and on Part II of the interview, and to adjust for residual discrepancies between sample and population distributions on a range of socio-demographic variables. Detailed descriptions of weighting methods are presented elsewhere [28]. Variances were computed using Taylor series linearization [49], and statistical significance was evaluated using two-sided design-based tests at the 0.05 level of significance.

## Results

### Prevalence of personality disorders clusters

The prevalence of any PD in the Sao Paulo Metropolitan Area was 6.8%. The most frequent subtype was Cluster C (4.6%), followed by Clusters A and B (4.3% and 2.7%, respectively) (Table 1).

**Table 1:** Prevalence estimates of personality disorders clusters in the Sao Paulo Metropolitan Area.

Personality disorders	%	SE
**Cluster A**	4.3%	0.7
**Cluster B**	2.7%	0.5
**Cluster C**	4.6%	0.7
**Any personality disorder**	6.8%	1.0

SE: standard error

### Sociodemographic correlates

Men had increased odds of presenting a personality disorder (OR = 2.2) [95% CI = 1.2–4.2] and, more specifically, a Cluster A diagnosis (OR = 6.8) [95% CI = 2.4–19.1]. Education was inversely associated with Cluster C (OR = 0.7) [95% CI = 0.5–0.9]. Age, income, marital and employment status were not significant predictors (Table 2).

**Table 2:** Sociodemographic predictors of personality disorders in the Sao Paulo Metropolitan Area.

	Cluster A	Cluster B	Cluster C	Any personality disorder
	OR (95% CI)	OR (95% CI)	OR (95% CI)	OR (95% CI)
**Gender**				
** Female**	1.0	1.0	1.0	1.0
** Male**	6.8 (2.4–19.1)**	1.3 (0.7–2.6)	1.2 (0.6–2.4)	2.2 (1.2–4.2)*
**Age**	0.8 (0.6–1.1)	0.7 (0.5–1.1)	1.2 (0.9–1.7)	0.9 (0.6–1.2)
**Education**	0.9 (0.6–1.2)	0.8 (0.6–1.2)	0.7 (0.5–0.9)*	0.8 (0.5–1.3)
**Income**	0.9 (0.6–1.5)	0.9 (0.4–1.7)	0.8 (0.6–1.3)	0.8 (0.5–1.4)
**Marital Status**				
** Married or cohabiting**	0.7 (0.3–1.6)	0.5 (0.2–1.2)	1.2 (0.4–3.5)	0.8 (0.3–1.9)
**Previously married**^**a**^	0.6 (0.2–2.0)	1.0 (0.4–2.5)	1.5 (0.5–4.6)	0.8 (0.3–2.1)
** Never married**	1.0	1.0	1.0	1.0
**Employment**				
** Employed/Student/Homemaker**	0.6 (0.2–1.5)	0.5 (0.2–1.3)	0.7 (0.2–2.1)	0.6 (0.3–1.2)
** Retired**	0.4 (0.1–1.8)	0.2 (0.0–2.4)	0.6 (0.3–1.1)	0.5 (0.1–1.8)
**Other**^**b**^	1.0	1.0	1.0	1.0

^a^ Separated, divorced or widowed

^b^ Unemployed or disabled

OR (95% CI): Odds ratio (95% confidence interval)

* p < .05

**p < .01

***p < .001

### Urban stressors

After adjustment for age, sex and all urban stressors simultaneously, there was no significant difference between being raised on the rural zone, a small town or a large city on the prevalence of personality disorders. Migration status was not a significant predictor either, as well as currently living in a socially deprived neighborhood (Table 3).

**Table 3:** Association of personality disorders with urban stressors in the Sao Paulo Metropolitan Area, adjusted for sex, age and all urban stressors simultaneously.

	Cluster A	Cluster B	Cluster C	Any personality disorder
	OR (95% CI)	OR (95% CI)	OR (95% CI)	OR (95% CI)
**Urbanicity**				
** Rural areas**	1.0	1.0	1.0	1.0
** Small town**	1.1 (0.4–3.1)	1.3 (0.5–3.6)	1.1 (0.4–2.8)	1.2 (0.5–3.0)
** Large cities**	1.2 (0.4–3.8)	0.9 (0.2–3.6)	0.8 (0.3–2.5)	1.0 (0.4–2.5)
**Migration status**				
** Non-migrants**	1.0	1.0	1.0	1.0
** Migrants**	1.2 (0.5–3.4)	1.0 (0.2–3.9)	0.9 (0.4–2.0)	1.1 (0.5–2.6)
**Neighborhood Social Deprivation**				
** No-low NSD**	1.0	1.0	1.0	1.0
** Low-medium/medium NSD**	1.0 (0.4–2.9)	0.9 (0.3–2.6)	1.1 (0.5–2.5)	1.1 (0.4–2.9)
** High/very high NSD**	1.3 (0.6–3.1)	1.3 (0.4–4.2)	1.4 (0.6–3.0)	1.4 (0.5–4.0)
**Number of crime-related traumatic events**				
** 0**	1.0	1.0	1.0	1.0
** 1**	1.6 (0.7–3.5)	1.3 (0.6–3.1)	2.0 (1.0–3.9)	1.5 (0.8–3.0)
** 2**	2.3 (0.8–6.8)	2.2 (0.7–7.2)	2.0 (1.0–4.0)	1.7 (0.6–4.3)
** 3 or more**	3.8 (0.9–16.6)	5.1 (1.8–14.7)**	3.5 (1.5–8.5)**	3.3 (1.6–7.0)**

OR (95% CI): Odds ratio (95% confidence interval)

* p < .05

**p < .01

***p < .001

However, with the exception of Cluster A, personality disorders were associated with cumulative exposure to crime-related traumatic experiences and this respected a dose-response gradient. Those with a history of three or more events had increased odds of Cluster B (OR 5.1) [95% CI = 1.8–14.7), Cluster C (OR 3.5) [95% CI = 1.5–8.5] and any personality disorder (OR 3.3) [95% CI = 1.6–7.0].

### Co-occurrence with other 12-month mental disorders

Personality pathology frequently co-occurred with other mental disorders. Almost two thirds of individuals with any PD had at least one co-occurring diagnosis, being anxiety the most common condition (46.3%). Cluster B had the highest prevalence of any co-occurrence (83.8%), followed by Clusters C (73.4%) and A (54.0%). Anxiety disorders were the most common co-occurrence of Clusters A and C (33.9% and 60.0%, respectively), while mood disorders predominated in Cluster B (48.8%) (Table 4).

**Table 4:** Prevalence of other 12-month mental disorders among those with personality disorders in the São Paulo Metropolitan Area.

12-month disorders	Cluster A	Cluster B	Cluster C	Any Personality Disorder
	% (SE)	% (SE)	% (SE)	% (SE)
**Any anxiety disorder**	33.9% (7.4)	47.9% (8.5)	60.0% (6.9)	46.3% (6.8)
**Any mood disorder**	23.3% (5.6)	48.8% (9.4)	42.1% (6.0)	31.7% (5.5)
**Any impulse disorder**	19.4% (5.1)	24.4% (6.3)	14.4% (4.1)	16.4% (4.3)
**Any substance disorder**	13.9% (4.3)	45.2% (9.0)	10.6% (3.3)	14.3% (3.6)
**Number of disorders**				
** Exactly one**	20.1% (5.6)	22.0% (5.5)	20.8% (4.5)	21.2% (4.8)
** Exactly two**	14.2% (4.1)	16.0% (5.7)	19.0% (4.8)	14.8% (4.4)
** Three or more**	19.7% (5.4)	45.7% (7.7)	33.6% (5.5)	27.4% (5.1)
**Any 12-month disorder**	54.0% (8.1)	83.8% (9.3)	73.4% (6.2)	63.4% (8.2)

SE: standard error

According to Table 5, personality disorders predict the occurrence of other 12-month diagnoses. Those with any PD were six times as likely to have one or more comorbid mental disorders (OR = 6.0) [95% CI = 2.6–14.0]. The strength of the associations respected a dose-response gradient (ORs for exactly one, two and three or more disorders were 3.1, 6.9 and 17.1, respectively) [95% CIs = 1.2–7.7; 2.1–22.6; and 6.4–57.9, respectively). Cluster B had the strongest association with any comorbidity (OR = 16.0) [95% CI = 3.6–70.8]. Cluster A was mostly associated with impulse-control disorders (OR = 6.1) [95% CI = 2.6–14.0]; Cluster B, with substance disorders (OR = 32.0) [95% CI = 10.7–95.7]; and Cluster C, with anxiety disorders (OR = 8.1) [95% CI = 4.0–16.2] (Table 5).

**Table 5:** Associations of personality disorders with other 12-month mental disorders in the Sao Paulo Metropolitan Area.

Personality disorders	Subtypes of 12-month mental disorders					Number of 12-month mental disorders		
	Any anxiety disorder	Any mood disorder	Any impulse disorder	Any substance disorder	Any 12-month disorder	Exactly one	Exactly two	Three or more
	OR (95% CI)	OR (95% CI)	OR (95% CI)	OR (95% CI)	OR (95% CI)	OR (95% CI)	OR (95% CI)	OR (95% CI)
**Cluster A**	3.4 (1.5–7.4)**	4.0 (1.9–8.4)**	6.1 (2.6–14.0)***	3.0 (1.1–7.8)*	4.4 (2.1–9.1)***	2.6 (1.0–6.6)*	5.6 (2.3–13.5)***	8.5 (3.1–23.2)***
**Cluster B**	4.4 (2.0–9.8)**	9.4 (3.9–22.6)***	7.2 (3.3–15.8)***	32.0 (10.7–95.7)***	16.0 (3.6–70.8)**	6.7 (1.6–28.6)*	14.5 (2.2–96.3)*	51.6 (11.2–238.7)***
**Cluster C**	8.1 (4.0–16.2)***	7.7 (4.4–13.7)***	4.6 (2.3–9.4)***	3.6 (1.7–7.8)**	8.8 (4.4–17.3)***	3.8 (1.7–8.4)**	11.6 (4.6–29.0)***	26.8 (12.4–57.9)***
**Any PD**	5.2 (2.8–9.8)***	5.4 (2.8–10.4)***	5.1 (2.4–10.7)***	4.2 (1.9–9.5)**	6.0 (2.6–14.0)**	3.1 (1.2–7.7)*	6.9 (2.1–22.6)**	17.1 (6.4–45.7)***

Odds ratio (OR) and 95% CI were estimated using multiple imputation logistic regression models in which personality disorders were treated as predictors and other 12-month mental disorders were treated as outcomes. Models were adjusted for gender and age

* p < .05

**p < .01

***p < .001

### Impairment associated with personality disorders

Personality disorders are associated with significant impairment in the previous month, as shown in Table 6. In the first model, adjusted for gender and age, the presence of any PD predicted global impairment and specific impairments in cognition, mobility, role functioning and social interaction, but not on self-care. Similar patterns could also be found among those with Cluster B or C, but not with Cluster A, which had less associated impairment.

**Table 6:** Impairment associated with personality disorders in the Sao Paulo Metropolitan Area.

	WHO-DAS					
	Self-care	Cognition	Mobility	Role functioning	Social interaction	Global
	β (SE)	β (SE)	β (SE)	β (SE)	β (SE)	β (SE)
**MODEL 1**						
** Cluster A**	0.2 (0.1)	0.5 (0.2)	0.1 (0.1)	0.3 (0.2)*	0.3 (0.2)	0.4 (0.2)*
** Cluster B**	0.5 (0.2)	0.9 (0.3)**	0.3 (0.2)	0.6 (0.2)*	0.8 (0.3)*	0.8 (0.2)**
** Cluster C**	0.3 (0.2)	0.9 (0.2)**	0.4 (0.2)*	0.7 (0.2)**	0.8 (0.3)**	0.9 (0.2)**
** Any PD**	0.2 (0.1)	0.7 (0.2)**	0.2 (0.1)*	0.5 (0.2)*	0.5 (0.2)*	0.6 (0.2)**
** Any MD**	0.2 (0.0)***	0.5 (0.1)***	0.3 (0.1)***	0.5 (0.1)***	0.4 (0.1)***	0.6 (0.1)***
**MODEL 2**						
** Cluster A**	0.1 (0.1)	0.3 (0.2)	0.0 (0.1)	0.2 (0.1)	0.2 (0.2)	0.2 (0.2)
** Cluster B**	0.4 (0.2)	0.7 (0.3)*	0.1 (0.2)	0.3 (0.2)	0.7 (0.3)*	0.5 (0.2)*
** Cluster C**	0.2 (0.2)	0.7 (0.2)*	0.3 (0.2)	0.5 (0.2)*	0.7 (0.3)*	0.6 (0.2)**
** Any PD**	0.1 (0.1)	0.5 (0.2)*	0.1 (0.1)	0.3 (0.2)	0.4 (0.2)*	0.4 (0.2)*
** Any MD**	0.2 (0.0)***	0.5 (0.1)***	0.3 (0.1)***	0.5 (0.1)***	0.3 (0.1)***	0.5 (0.1)***

WHO-DAS: World Health Organization Disability Assessment Schedule; PD: personality disorder; MD: 12-month mental disorder

MODEL 1: adjusted for gender and age

MODEL 2: adjusted for gender, age and other 12-month mental disorders

* p < .05

**p < .01

***p < .001

In the second model, after adjusting for other 12-month diagnoses (in addition to gender and age), associations became weaker or non-significant. Any PD and Cluster B remained significantly associated with global, cognition and social interaction impairments; and Cluster C, with cognition, role functioning and social interaction. Noteworthy, Clusters B and C were more strongly associated with cognition impairment than other 12-month mental disorders (**β** = 0.7, 0.7 and 0.5, respectively). Likewise, Clusters B and C were more strongly associated with social interaction impairment than other 12-month mental disorders (**β** = 0.7, 0.7 and 0.3, respectively). Global impairment was greater for Cluster C, when compared to other 12-month mental disorders (**β** = 0.6 and 0.6, respectively). Cluster A, however, was not shown to be specifically associated with impairment.

### Treatment

According to Table 7, approximately 20% of those with any PD had received treatment for emotional or substance use problems in the year previous to the interview. The prevalence of treatment in specialized mental health care was greater than that of general medical or non-health care (14.4% vs. 7.1% and 5.6%, respectively). Treatment was more prevalent among those with Cluster C, followed by Clusters B and A, in all care modalities.

**Table 7:** Prevalence of 12-month treatment among those with personality disorders in the Sao Paulo Metropolitan Area.

Personality disorders		Health care					Non-health care		
	Any treatment	Mental Health Specialist							
		Psychiatrist	Other mental health^§^	Any mental health	General medical^‡^	Any health care	Human service^||^	CAM^¶^	Any non-health care
	% (SE)	% (SE)	% (SE)	% (SE)	% (SE)	% (SE)	% (SE)	% (SE)	% (SE)
**Cluster A**	15.4 (4.6)	8.2 (3.5)	5.4 (2.8)	11.3 (4.4)	5.3 (3.0)	13.2 (4.5)	2.8 (2.0)	3.6 (2.5)	5.3 (2.7)
**Cluster B**	22.6 (5.6)	11.2 (3.9)	7.9 (3.5)	16.0 (5.5)	8.0 (3.3)	18.4 (5.6)	2.8 (1.9)	4.9 (2.9)	7.1 (3.3)
**Cluster C**	26.7 (5.1)	13.3 (3.8)	8.4 (3.3)	17.9 (4.8)	8.1 (2.9)	22.7 (5.1)	3.6 (2.3)	5.4 (2.7)	7.9 (3.0)
**Any PD**	20.0 (4.1)	10.9 (3.0)	6.9 (2.5)	14.4 (3.4)	7.1 (2.7)	18.0 (4.1)	2.9 (1.6)	3.9 (2.2)	5.6 (2.5)

SE: standard error; CAM: complementary and alternative medicine

^§^ defined as psychologists or other non-psychiatrist mental health professionals in any setting, social worker or counsellor in a MHS setting or use of a mental health hotline

^**‡**^ defined as a primary care physician, other general physician, nurse, and any other health professional not previously mentioned

^||^ defined as a religious or spiritual advisor or social worker or counsellor in any setting other than a specialty mental health setting

^¶^ defined as any other type of healer, participation in an Internet support group, or participation in a self-help group.

Subjects with personality disorders had increased odds of treatment in the previous year (OR 3.0) [95% CI = 1.6–5.5], regardless of gender and age. This association was stronger for consulting a psychiatrist rather than other mental health specialists or other general medical providers (OR 4.1 vs. 2.6 and 3.1, respectively) [95% CIs = 2.0–8.7; 1.0–6.5; and 1.1–8.2, respectively]. Remarkably, after adjustment for other 12-month mental disorders (in addition to gender and sex), only Cluster C remained significantly associated with treatment (OR 2.0) [95% CI = 1.0–3.8] (Table 8).

**Table 8:** Association between personality disorders and 12-month treatment in the Sao Paulo Metropolitan Area.

	Any treatment	Health care					Non-health care		
		Mental Health Specialist							
		Psychiatrist	Other mental health^§^	Any mental health	General medical^‡^	Any health care	Human service^||^	CAM^¶^	Any non-health care
	OR (95% CI)	OR (95% CI)	OR (95% CI)	OR (95% CI)	OR (95% CI)	OR (95% CI)	OR (95% CI)	OR (95% CI)	OR (95% CI)
**MODEL 1**									
**Cluster A**	2.5 (1.0–6.0)*	3.1 (1.0–9.7)	2.3 (0.6–8.9)	2.7 (0.9–8.2)	2.6 (0.5–13.4)	2.5 (0.9–6.8)	2.3 (0.3–17.7)	3.1 (0.5–20.7)	2.8 (0.8–9.7)
**Cluster B**	3.0 (1.4–6.2)**	3.7 (1.6–8.6)**	2.5 (0.8–7.6)	3.2 (1.2–8.1)*	2.9 (1.1–7.8)*	2.7 (1.2–6.3)*	1.9 (0.4–9.0)	3.8 (0.9–16.7)	3.2 (1.0–10.3)
**Cluster C**	3.8 (2.0–7.3)**	4.3 (1.7–10.9)**	2.8 (1.0–8.4)	3.7 (1.5–9.0)**	2.9 (1.2–7.1)*	3.5 (1.6–7.7)**	2.6 (0.6–10.7)	4.9 (1.4–17.0)*	4.0 (1.6–10.1)**
**Any PD**	3.0 (1.6–5.5)**	4.1 (2.0–8.7)**	2.6 (1.0–6.5)*	3.3 (1.7–6.3)**	3.1 (1.1–8.2)*	3.1 (1.6–6.1)**	2.3 (0.6–8.7)	3.1 (0.7–14.0)	2.8 (0.9–8.7)
**MODEL 2**									
**Cluster A**	1.5 (0.6–3.8)	1.7 (0.5–5.5)	1.4 (0.4–5.7)	1.6 (0.5–5.1)	1.5 (0.3–8.2)	1.5 (0.5–4.3)	1.4 (0.2–11.1)	1.9 (0.3–13.6)	1.7 (0.5–5.8)
**Cluster B**	1.4 (0.7–2.9)	1.5 (0.7–3.5)	1.3 (0.4–4.0)	1.5 (0.6–3.8)	1.4 (0.5–3.7)	1.3 (0.5–3.0)	1.0 (0.2–4.7)	1.9 (0.4–8.3)	1.5 (0.5–4.7)
**Cluster C**	2.0 (1.0–3.8)*	2.0 (0.8–5.1)	1.6 (0.5–4.7)	1.9 (0.8–4.6)	1.4 (0.6–3.5)	1.8 (0.8–3.9)	1.4 (0.3–6.2)	2.7 (0.7–9.9)	2.0 (0.8–5.3)
**Any PD**	1.7 (0.9–3.0)	2.1 (1.0–4.4)	1.5 (0.6–4.1)	1.8 (0.9–3.5)	1.6 (0.6–4.5)	1.7 (0.9–3.4)	1.3 (0.3–5.4)	1.8 (0.4–8.3)	1.5 (0.5–7.7)

OR (95% CI): Odds ratio (95% confidence interval); CAM: complementary and alternative medicine

^§^ defined as psychologists or other non-psychiatrist mental health professionals in any setting, social worker or counsellor in a MHS setting or use of a mental health hotline

^**‡**^ defined as a primary care physician, other general physician, nurse, and any other health professional not previously mentioned

^||^ defined as a religious or spiritual advisor or social worker or counsellor in any setting other than a specialty mental health setting

^¶^ defined as any other type of healer, participation in an Internet support group, or participation in a self-help group

model 1: adjusted for gender and age; model 2: adjusted for gender, age and other 12-month mental disorders

* p < .05

**p < .01

***p < .001

## Discussion

Personality disorders are common in the Sao Paulo Metropolitan Area. They affect 6.8% of its adult residents, with a predominance of Cluster C. They are significantly associated with other mental disorders and predict functional impairment, especially in the cognition and social interaction domains. These subjects have increased odds of receiving treatment for emotional and substance use problems, but associations become largely non-significant when adjusted for other 12-month psychopathology.

### Prevalence

The prevalence estimates of PDs in the Greater Sao Paulo are within the ranges found in previous epidemiological surveys for any PD (6.8% vs. 4.4–13.4%); Cluster A (4.3% vs. 1.6–5.7%), which includes paranoid, schizoid and schizotypal PDs; Cluster B (2.7% vs. 1.2–5.5%), encompassing antisocial, borderline, histrionic and narcissistic PDs; and Cluster C (4.6% vs. 2.3–9.4%), which includes avoidant, dependent and obsessive-compulsive PDs [3–9]. As seen, estimates vary markedly from one study to another, and this might be due to random variance, cross-cultural or methodological differences.

In order to allow more clear-cut transcultural comparisons, countries participating of the WMH adopted similar sampling, data collection and statistical methods. A cross-national analysis including low-, middle- and high-income countries from different WHO regions estimated the prevalence of any PD at 6.1%. Cluster A disorders predominated (3.6%), followed by Clusters C (2.7%) and B (1.5%) [30]. Since Sao Paulo Megacity participates in this consortium, our estimates may be directly compared to those findings. While the prevalence of any PD in our study was quite similar to the cross-national mean (6.8% vs. 6.1%), specific clusters showed different patterns. The prevalence of Cluster A in Sao Paulo (4.3%) was higher than the overall mean in the WMH (3.6%), but lower than other Latin American countries (5.3% in Colombia, and 4.6% in Mexico). On the other hand, the prevalence of Clusters B and C in our study was higher than all other WMH sites.

### Sociodemographic correlates

Recognizing sociodemographic correlates may help identify those in greater risk of PDs, and help tailor adequate interventions. It may also shed light on possible causal mechanisms and help advance physiopathological understanding.

In our study, men had a greater chance of presenting PD and, more specifically, a Cluster A diagnosis, what is consistent with the literature [4–6, 9, 30]. Other authors have observed an association of being male with Clusters B [4, 6] and C [30]. Some studies, however, did not observe such gender differences [3, 7].

We also found an association between Cluster C and lower educational attainment, and this possibly reflects the negative impact of anxious and fearful traits on education.

We did not observe significant differences regarding age, income, marital or employment status, and this may be due to lack of enough statistical power. Previous studies have shown that PDs, especially Cluster B, are more common in the young [3, 4, 6]. Likewise, the literature indicates that PDs are more prevalent among the separated, divorced or never married, as well as the unemployed and those with lower income [50].

### Association with urban stressors

Urbanicity was not shown to significantly influence the occurrence of PDs. Previous studies have compared the prevalence of PDs in residents of city centers, metropolitan areas, suburbs and countryside [4, 7, 51–54]. They have focused, however, on present place of residence, rather than the impact of being born and raised in urban areas. Therefore, the influence of urbanicity on PDs remains to be further explored in future research.

Migration was not a significant predictor as well, although some authors suggest that migrants are at increased risk [55]. This perspective is not consensual, however, and some studies indicate a lower prevalence of PDs in immigrants admitted to psychiatric emergency services in developed countries [56–58]. Still, no solid conclusion can be drawn from these clinical samples since immigrants and natives may have differential probabilities of searching for mental health services.

Living in socially-deprived neighborhoods did not have any impact on personality pathology either. This may be the result of the design of the SPMHS, that assessed only current residency, and not the effect of social deprivation throughout life.

From all urban stressors investigated, only cumulative exposure to violence was associated with Clusters B and C and any personality disorder. Violence is endemic in the Sao Paulo Megacity [17], and repetitive crime victimization may alter neurobiological, psychological, interpersonal and social mechanisms associated with normal personality functioning. In this sense, recurrent exposure to crime may amplify the expression of the dramatic, emotional or erratic traits associated with Cluster B PDs, and of the anxious or fearful traits associated with Cluster C PDs. Recurrent exposure to violence, thus, would be a possible explanation to the highest prevalence of Clusters B and C in the Sao Paulo Megacity in comparison to other sites of the WMH consortium. This hypothesis needs further exploration.

### Co-occurrence

Personality disorders were highly co-occurrent with other 12-month mental disorders, confirming findings from previous research conducted in diverse sites and populations [3, 4, 6–9, 25, 30]. These associations were especially strong for Cluster B and respected a dose-response gradient.

Investigating psychiatric comorbidity is a reflection on the very nature of psychopathology and current taxonomies. Why do personality and mental disorders co-occur? Diverse models have been proposed, such as the pathoplastic (one may influence the appearance or presentation of the other); the spectrum (they may share a common etiology); and the causal model (one may have a causal role in the etiology of the other) [59]. Testing these models is beyond the scope of this study, but some initiatives have been trying to disentangle these relationships. One is the Hierarchical Taxonomy of Psychopathology (HiTOP), which aims to explore the structure of mental disorders by means of dimensional measures of psychopathology [60].

Apart from etiological and taxonomical considerations, an important aspect regarding co-occurrence is its clinical impact, which reflects on functioning and treatment.

### Functional impairment

PDs were associated with significant functional impairment. These are chronic conditions that, even when not comorbid, have negative effects on functioning. Interestingly, “pure” personality pathology may be even more impairing than other mental disorders. “Pure” Cluster C, for example, was more strongly associated with global impairment than other 12-month mental disorders. These results raise the question why formerly-known as “Axis I” disorders still receive greater clinical and research attention.

### Treatment

Lack of treatment of mental disorders is a common reality in the Sao Paulo Metropolitan Area [25]. This is especially true for personality pathology, as only one in five cases received treatment in the previous year.

With the exception of Cluster C, “pure” PDs remained largely undertreated, in spite of direct functional impairment. Several barriers may account for this situation, and possibly the primary ones are lack of recognition, misunderstanding and stigma. Personality disorders are often ego-syntonic [61] and many people are unaware of their own pathological traits [62, 63]. Regarding families, misunderstanding and misinformation is frequent even among relatives of diagnosed patients [64]. Altogether, these factors may limit treatment need awareness.

Even after overcoming these initial barriers, subjects face additional obstacles on the healthcare system. Access to treatment is unequal, resources in the public health system are insufficient and misallocated, general practitioners are not adequately trained in mental health, the provision of specialized care is limited and services are not adequately integrated [65–67]. Stigma against personality disorders is also a common phenomenon among health care providers. PD assessment is often regarded as time consuming, difficult and not based on specific criteria, and these patients are frequently labeled as difficult, non-adherent and refractory [68, 69]. In our study, treatment was predominantly associated with comorbid, rather than “pure” PDs. This may be the result of greater recognition and less stigma against previously-called “Axis I”. Comorbidity may also result in greater clinical severity and, thus, increased treatment search and receipt. Noteworthy, Cluster C remained significantly associated with treatment after adjustment of other mental disorders. Compared to other Clusters, these individuals may be more self-conscious and insightful, less denying and more prone to adopt health-related behaviors. Although Cluster B is traditionally regarded as the most troublesome group of PDs, a central feature is their being erratic and impulsive, which may limit their access to treatment. And Cluster A individuals, for their eccentric and isolation characteristics, may have less insight and more treatment avoidance.

Comparing these results with the WMH cross-national analysis [30], treatment prevalence among subjects with PDs in Nigeria, a lower middle income country is 6.0%; treatment prevalence varies from 6.6 to 19.9% in upper middle income countries (Colombia, Lebanon, Mexico, People’s Republic of China and South Africa); and from 21.6 to 37.3% in high income countries (Western Europe and USA, respectively). In this sense, although Brazil is an upper middle income country[70], treatment prevalence among PD subjects in the Sao Paulo Metropolitan Area (25%) is similar to high income countries. But this does not mean that subjects with PDs in the SPMA are in a privileged situation. A closer look may realize that lack of treatment is the rule, not the exception, among those with personality pathology, no matter the wealthy of the country of residency.

### Limitations

These results must be considered bearing in mind some potential limitations. Diagnoses were generated from IPDE screening questions using equations derived from the US clinical reappraisal sample. Although prediction accuracy was considered excellent, no other WMH country directly calibrated IPDE diagnoses. Furthermore, empirical studies about the three-cluster model of PDs have shown mixed results [71–74]. Some concern is also raised by the cross-sectional nature of our data, which precludes any conclusion about direction of associations or causality.

## Conclusion

This study may offer some relevant contributions to scientific knowledge, to public health policies and to clinical practice.

As indicated in the introduction, little is known about the epidemiology of personality disorders in LMIC. These results help bridge this gap and may reflect what happens in other megacities of the developing world.

In our study, violence was associated with increased odds of personality pathology, but other urban stressors were not. Probably the influence of urban life on personality is a more fine-grained phenomenon, and would be better explained by factors such as social disintegration [75], lack of social support and of shared values, interfering with family functioning and with buffering from the social community [76]. In 1975, Fischer stated that “social networks are the internal fabric of the social structure” and “the possible link between individual and community” [77]. Future studies on the association of urban life and personality disorders would benefit from focusing on the impact of urban stressors on the dynamics of social networks in different developmental stages. This would be assessed by methods such as longitudinal network analysis.

Research is also needed in other LMIC. More studies would consolidate our understanding about the prevalence, the specificities and communalities of PDs in the developing world. Ideally, these studies would have a prospective design and use not only categorical, but also dimensional measures of personality pathology.

Regarding public health, this study made clear the unmet needs of subjects with PDs. This group would benefit from educational interventions directed to lessen stigma and improve identification of cases. Services must be adequately structured to accommodate these patients, and educational programs must be offered not only to specialized but also to general practitioners.

Our data may also be relevant to clinical practice. Formerly-known as “Axis I” disorders have been the main focus of clinical attention on the last decades. This is probably the result of stigma and misbeliefs about PD patients as difficult, malingering, non-adherent and untreatable. These are indeed complex clinical conditions, sometimes more impairing than other mental disorders. With better training, however, practitioners may start feeling more capable of understanding, diagnosing and treating these patients. They may start to decode patients’ apparent “being difficult” as a symptom of their disorder. They may start to wonder what may have happened in their lives for their personalities to be like this. And then they may finally become able of sustaining a therapeutic relationship with these patients, the most important element in the treatment of personality disorders.

## Supporting information

S1 Fig Steps involved in the multiple imputation of personality disorders diagnoses from IPDE screening questions. DS 1–10: datasets 1 to 10; PE 1–10: parameter estimates 1 to 10.
(PDF)

S1 Dataset Dataset with variables used in this study. (XLS)
