# Supplementary figures and images for: The epidemiology of personality disorders in the Sao Paulo Megacity general population
Source: PLoS One. 2018 Apr 24;13(4):e0195581. doi: 10.1371/journal.pone.0195581 (PMC5978986; doi:10.1371/journal.pone.0195581)

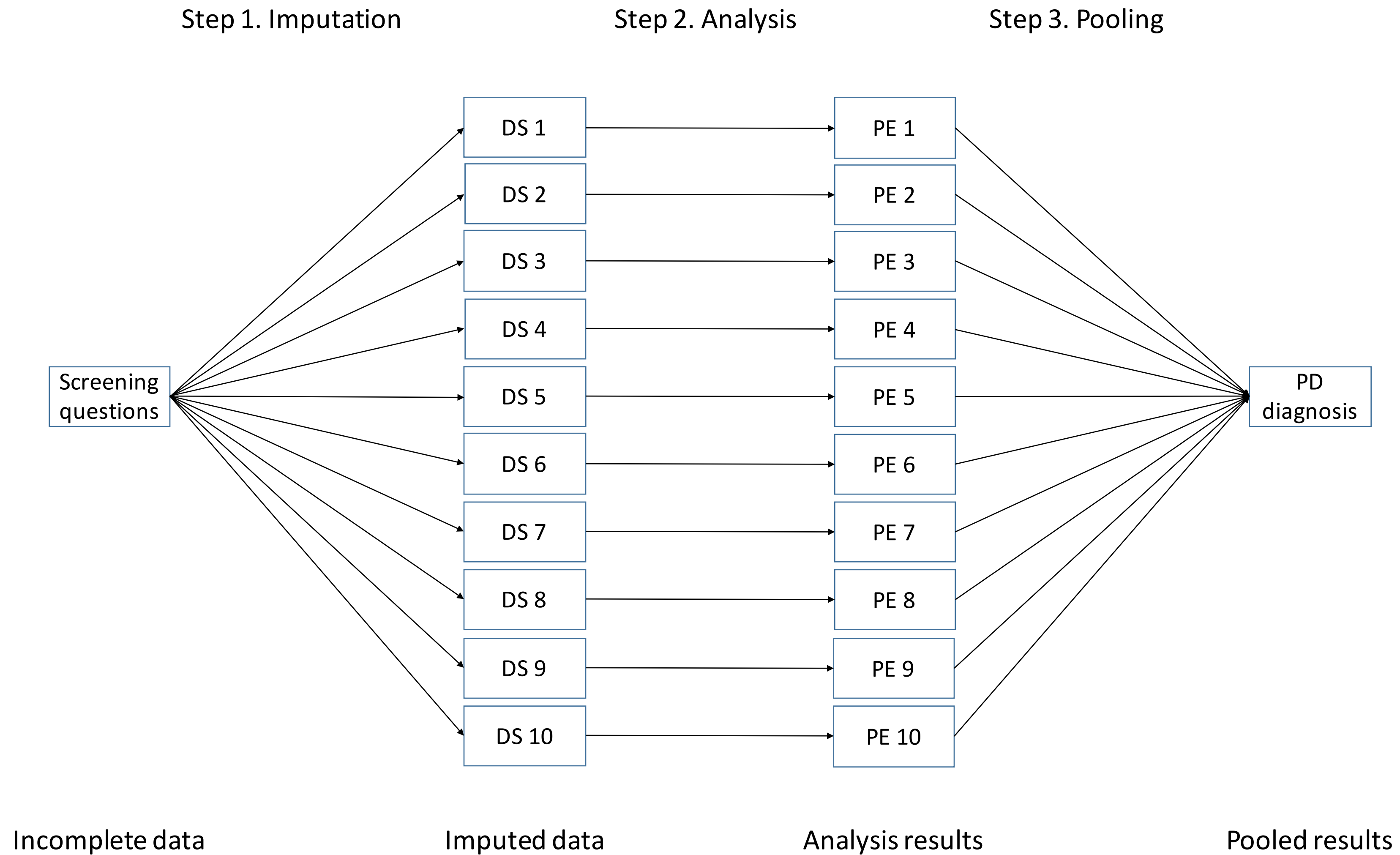

Supplement: S1 Fig — DS 1–10: datasets 1 to 10; PE 1–10: parameter estimates 1 to 10. (PDF) [file pone.0195581.s001.pdf]
